# The relationship between carotid disease and retinopathy in diabetes: a systematic review

**DOI:** 10.1186/s12933-020-01023-6

**Published:** 2020-05-06

**Authors:** Jocelyn J. Drinkwater, Timothy M. E. Davis, Wendy A. Davis

**Affiliations:** Medical School, The University of Western Australia, Fremantle Hospital, P. O. Box 480, Fremantle, WA 6959 Australia

**Keywords:** Diabetes mellitus, Diabetic retinopathy, Carotid disease, Carotid intima-media, Carotid plaque, Carotid atherosclerosis

## Abstract

**Background:**

Since studies of the relationship between carotid disease and diabetic retinopathy (DR) have shown apparent inconsistencies, the aim of this study was to conduct a systematic review of available published data.

**Methods:**

Electronic databases were searched independently by two reviewers, according to an iterative protocol, for relevant articles. The search term used was “diabetes AND (carotid disease OR intima-media OR carotid plaque OR carotid stenosis OR carotid arterial disease OR carotid artery disease OR carotid atherosclerosis) AND (retinopathy OR diabetic retinopathy)”.

**Results:**

From 477 publications, 14 studies were included. There were differences in the variables used as markers of carotid disease and DR across the included studies. Ten studies used carotid disease as the dependent variable, and the remainder used DR. All but one study involved cross-sectional data. Most studies reported a statistically significant association between at least one parameter of carotid disease as assessed by ultrasound and DR presence or severity. Only four studies reported no significant association. A common limitation was the use of convenience participant sampling.

**Conclusions:**

There appears to be an increased likelihood of DR when there is ultrasonographic evidence of carotid disease, and vice versa. The available studies suggest that there may be a direct relationship between DR and carotid macrovascular disease and/or that these complications co-exist due to shared risk factors. If carotid disease is detected, retinal assessment should be performed. If DR is identified, intensive cardiovascular disease risk management should be considered. Additional longitudinal studies are needed to assess the directionality of the association.

## Background

Diabetic retinopathy (DR) is a common microvascular complication affecting over one-third of people with diabetes mellitus [1]. It is one of the leading causes of moderate or severe visual impairment globally [2]. Known modifiable risk factors comprise hyperglycemia, hypertension and dyslipidemia [1, 3], but the pathophysiology of DR is not fully understood. Several biochemical mechanisms have been suggested including increased oxidative stress, inflammation, neurodegeneration and upregulation of vascular endothelial growth factor (VEGF) [4]. Despite screening programs aimed at early detection, there are few treatments for DR and most can only be used to prevent worsening of established disease [5]. There is, therefore, a need for improved understanding of the epidemiology of DR including risk factors so that it can be identified earlier, new preventive strategies and treatments developed, and vision loss prevented or delayed.

Diabetic retinopathy is characterised by vascular lesions including microaneurysms, hemorrhages and exudates [4]. Less severe DR typically refers to mild to moderate non-proliferative diabetic retinopathy (NPDR), while more severe DR generally comprises severe NPDR or proliferative diabetic retinopathy (PDR) which have a greater effect on vision [4, 6]. The Early Treatment Diabetic Retinopathy Study protocol is commonly used to assess the severity of DR [6–10]. According to this scheme, mild NPDR consists of at least one microaneurysm without evidence of other pathological lesions [6]. When NPDR progresses to PDR there is evidence of neovascularisation [6].

Appropriately intensive management of hyperglycemia and hypertension reduces the risk of DR [3, 4]. Of the few available specific treatments, intravitreal anti-VEGF agents, such as aflibercept, ranibizumab and bevacizumab, are now considered as a first-line treatment for vision-threatening DR but not all patients respond and these therapies are costly [5, 11]. Laser photocoagulation, which used to be the first-line treatment, and intravitreal corticosteroids are also used to treat severe DR [3–5, 11]. In type 2 diabetes (T2D), fenofibrate is a treatment that can prevent DR progression. The Fenofibrate Intervention and Event Lowering in Diabetes (FIELD) study, which recruited 9795 participants, showed fenofibrate reduced the need for laser treatment and, in those with pre-existing DR, the risk of progression [12]. The Action to Control Cardiovascular Risk in Diabetes (ACCORD) Eye study, which involved 10,251 participants, showed that those in the fenofibrate and statin arm had lower odds of DR progression after 4 years than those in the placebo and statin only arm [13]. Nevertheless, there is still a residual risk of DR progression despite these interventions.

Carotid arterial disease is a major macrovascular complication of diabetes. It is generally assessed by ultrasound which can detect atherosclerotic plaque and quantify intima-media thickness (IMT; see Additional file 1: Figure S1) [14, 15]. The Mannheim consensus considers plaque to be a focal structure that encroaches into the arterial lumen of at least 0.5 mm or 50% of the surrounding IMT value, or demonstrates a thickness of ≥ 1.5 mm as measured from the media-adventitia interface to the intima-lumen interface [16]. The degree of stenosis associated with a plaque is expressed as a percentage of the lumen diameter. The IMT can be measured in the common carotid artery (CCA), at the bifurcation and in the internal carotid artery (ICA) [16]. Carotid disease is associated with stroke and cardiovascular disease [14, 15]. A review of six studies found an increased risk of cardiovascular events in people with a higher CCA IMT [17]. The presence and number of plaques have also been associated with greater risk of cardiovascular disease, but the combination of both increased CIMT and plaque may be a better measure [18]. People with an IMT ≥ 1 mm and those with high grade stenosis are considered to be at very high risk of cardiovascular events [14].

Microvascular disease in diabetes has been associated with macrovascular disease, a relationship that may reflect shared risk factors [19]. However, the specific association between carotid arterial disease as a manifestation of proximal macrovascular disease and DR as a form of microangiopathy is not well established. As the carotid artery supplies blood to the retina, it is possible carotid disease has a direct effect on the development of DR. The aim of the present systematic review was, therefore, to assess the evidence linking carotid disease and DR in people with diabetes.

## Methods

### Search strategy and selection criteria

An iterative protocol developed according to the Preferred Reporting Items for Systematic Review and Meta-Analysis (PRISMA) Statement was followed [20]. The electronic databases Embase, PubMed and MEDLINE were searched. The Boolean search term used was “diabetes AND (carotid disease OR intima-media OR carotid plaque OR carotid stenosis OR carotid arterial disease OR carotid artery disease OR carotid atherosclerosis) AND (retinopathy OR diabetic retinopathy)”. Studies were eligible for inclusion if they specifically assessed the relationship between carotid disease and DR using statistical analyses appropriate to the study design with relevant results clearly displayed and an effect size given. In cases where studies assessed the relationship but did not give effect sizes or clear results, the authors were contacted and studies were included if this information was provided. A minimum sample size of 50 participants (to reduce likelihood of type II error) and a statistical analysis that, as a minimum, assessed the effect of age and sex on the association between carotid disease and DR were required. Studies were restricted to those reported in English and conducted in adults. Those not specifying diabetes type and the criteria for determining carotid disease and DR were excluded. In vitro and animal studies, case studies, case series and conference abstracts were also excluded. The search included articles published to end-2019. The summary measures used were those reported in individual studies.

Two reviewers (JD and WD) independently conducted the literature search and reviewed all the articles assessing eligibility and extracting required information. The data from included studies were entered into pre-defined fields including the country where the research was conducted, year(s) the data were collected, study design, aims, definitions, type of diabetes, sample size, methods, results, covariates and limitations. The reference lists of the included articles were reviewed to ensure articles that met the inclusion criteria were not inadvertently excluded due to search terms. The risk of bias of each study was assessed according to guidelines in the Cochrane handbook [21]. The quality of the individual studies was assessed using the National Heart, Lung, and Blood Institute Quality Assessment Tool for Observational and Cross-Sectional Studies [22]. Although a meta-analysis was considered, this was not appropriate.

## Results

**Fig. 1:**
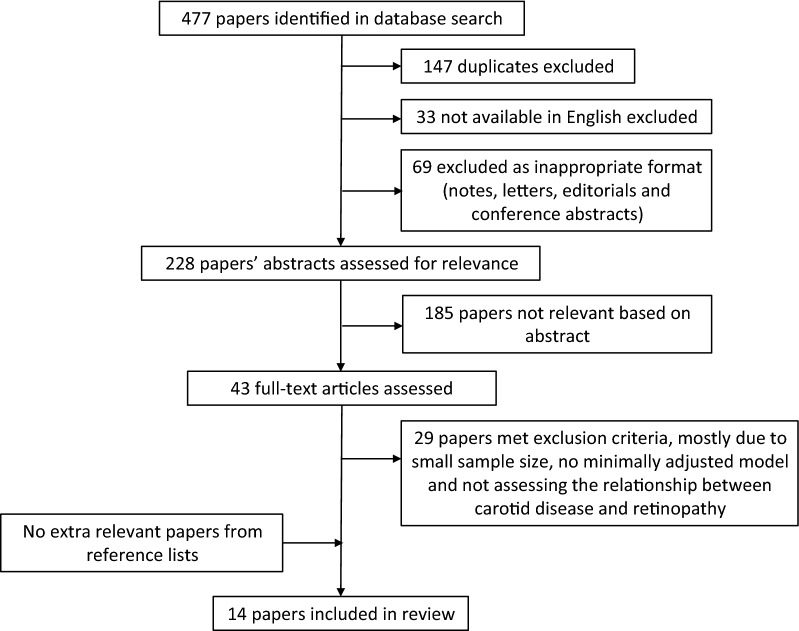
Consort diagram showing papers included in review

The process of article selection is summarised in Fig. 1. The search identified 477 publications. After excluding duplicates, those not available in English and those of inappropriate format (notes, editorials etc.), 228 abstracts were assessed for relevance and 43 full-text articles were suitable for further evaluation. We emailed authors of four publications for further information, but only received a response from one [23] and excluded the three studies for which no response was received [24–26]. No further papers were identified from checking reference lists of relevant papers. The final number of journal articles included in the review was 14 (see Fig. 1).

These 14 publications were from 10 different countries including three from South Korea [10, 27] and two each from Spain [7, 28] and Brazil [23, 29]. The Spanish papers were from the same study but one included participants with type 1 diabetes (T1D) [28] while the other included people with T2D [28]. The Brazilian publications were also from the same study sample, the Rio de Janiero Type 2 Diabetes Cohort Study (RDJ), but assessed different outcomes and so both publications were included [23, 29]. Most of the studies were cross-sectional and only one (the RDJ) included longitudinal data [23]. The characteristics of the included studies are shown in Table 1. The sample size ranged from 87 to 1607 participants. Most study samples comprised people with (T2D) but three included participants with T1D [7, 30, 31].

**Table 1:** Characteristics of included studies

First author name	Study name	Year published	Year(s) data collected	Country	Study design	Sample Size	Diabetes type	Age (years)	Females (%)	Diabetes duration (years)
Cardoso [23]	Rio de Janeiro Type 2 Diabetes Cohort Study	2019	Baseline: 2004–2008 follow up: 2017	Brazil	Prospective cohort study	478	2	60 ± 9.1	64.0	8 [3–15]
Hjelmgren [32]	Western Region initiative to Gather Information on Atherosclerosis (WINGA) Database	2019	2004–2010	Sweden	Cross-sectional analysis of retrospective data	445	2	68.9 ± 9.6	35.3	7.9 ± 7.0
Ichinohasama [8]		2019		Japan	Cross-sectional	129	2	54.0 [40.5–64.5] in no DR group, 55.5 [43.0–63.5] in mild NPDR group	41.8	6.0 [2.0–11.5] in no DR group, 11.0 [6.3–15.8] in mild NPDR group
Carbonell [7]		2018		Spain	Cross-sectional	340	1	45.0 [37.0–53.0]	54.4	20 [14–29]
Liu [34]	Meilong Town Diabetes Health Management Program	2015	2013	China	Cross-sectional community-based	1607	2	65.0 ± 7.6	55.3	9.9 [5.2–16.9] in DR group and 6.0 [2.9–11.0] in no DR group
Alonso [28]		2015		Spain	Cross-sectional	312	2	59.0 [48.5–66.0] in no DR group, 61.0 [54.0–68.0] in DR group	49.4	6 [2.5–10] in no DR group, 11 [6–20] in DR group
Jung [27]		2013	2009–2011	South Korea	Cross-sectional analysis of retrospective data	131	2	55.6 ± 11.3	45.8	7.4 ± 6.8
Cardoso [29]	Rio de Janeiro Type 2 Diabetes Cohort Study	2012	2004–2007	Brazil	Cross-sectional	441	2	60 ± 8.6	63.9	10.2 ± 8.6
Yun [10]		2011		South Korea	Cross-sectional	605	2	67.9 ± 9.8	69.8	8.7 ± 7.7
Son [10]		2011	2008–2010	South Korea	Cross-sectional	142	2	52.4 ± 13.6	31.7	Newly diagnosed
Araszkiewicz [30]		2011		Poland	Cross-sectional	87	1	34 [29–43]	50.6	10 [9–14]
Lacroix [33]		2006		France	Cross-sectional	300	2	62.5 ± 12.6	44.7	11 ± 9.2
Distiller [31]		2006		South Africa	Cross-sectional	148	1	48 (19–76)	48.6	26 (18–59)
Rema [9]	The Chennai Urban Rural Epidemiology Study (CURES-2)	2004		India	Cross-sectional population-based	590	2	52 ± 11	56.4	4 ± 5

Data presented as mean ± SD, median (range), percentage, median [inter-quartile range] or number. Some cells left blank intentionally as these data were not provided

There was heterogeneity between the studies included in our review. There were noticeable differences in the variables used as markers of carotid disease and DR, as described in Table 2. Measures used to assess carotid disease included measurement of intima media thickness (IMT), plaque, stenosis, or a combination of these. In addition to using different variables as markers of carotid disease, there were also differences in analyses as studies either used continuous variables, a clinically relevant level to split the data into two groups, or percentiles. Two studies assessed DR severity [7, 28] and the RDJ assessed new or worsening DR in their longitudinal analysis [23]. While most other studies determined the severity of DR in their participants, a dichotomous variable (DR absent or present) was used to assess the association with carotid disease. The methods by which DR was graded also varied between the studies. Four specifically stated that the ETDRS grading system was used and one used the International Clinical Diabetic Retinopathy Disease Severity Scale, but many did not give sufficient detail often simply stating that an ophthalmologist graded DR (see Table 2). Due to the significant methodological differences between these studies, a meta-analysis was not appropriate.

**Table 2:** Definitions used for carotid disease and diabetic retinopathy

First author	Diabetic retinopathy variable/s	Diabetic retinopathy assessment	Carotid disease variable/s	Carotid disease assessment
Cardoso [23]	New or worsening DR	DR graded as none, mild NPDR, moderate NPDR, severe NPDR or PDR by ophthalmologist at annual review. Worsening defined as worse by at least two grades (e.g. from mild to severe NPDR)	IMT of ICA, BIF and CCA Plaque score Number of plaques	Mean of 3 IMT measurements used, IMT measured according to Mannheim consensus Extracranial carotid artery plaque score assigned for each segment of ICA, CCA and BIF and highest grade assigned; 0: no plaque, 1: one small plaque, stenosis < 30%, 2: one medium plaque, 30–49% stenosis or multiple small plaques, 3: one large plaque, 50–99% stenosis or multiple plaques with at least one medium plaque, 4: 100% occlusion Single vascular radiologist performed ultrasound, good intra-observer test–retest reliability
Hjelmgren [32]	Any	Medical records from all eye clinics in the area	Stenosis > 50%	Greater stenosis from left or right artery used
Ichinohasama [8]	Mild NPDR versus no DR	Right eye assessed by ophthalmologist, according to ETDRS protocol	CCA IMT	Maximum measurement of right CCA IMT
Carbonell [7]	None, mild or advanced (moderate NPDR or worse)	Examination by ophthalmologist, according to ETDRS protocol	Plaque	Plaque was defined according to the Mannheim consensus Single sonographer at each study site performed ultrasound
Liu [34]	Any	Retinal images graded by ophthalmologist according to International Clinical Diabetic Retinopathy Disease Severity Scale	CCA IMT > 1 mm Plaque Subclinical atherosclerosis	Maximum CCA IMT value of left or right artery used, IMT was measured 1.5 cm proximal to the bifurcation, elevated CCA IMT defined as > 1 mm Plaque was classified as focal increase in thickness more than 0.5 mm or 50% of surrounding IMT Subclinical atherosclerosis was defined as CCA IMT > 1 mm and/or presence of carotid plaque Single sonographer performed ultrasound
Alonso [28]	Any and mild NPDR, moderate NPDR or severe NPDR or PDR	Multi-field stereoscopic retinal images and ophthalmologist examination	IMT of ICA, BIF and CCA Plaque	Semiautomatic software provided data for mean and mean-maximum IMT from segments of ICA, BIF and CCA. Values from the left and right arteries were averaged Plaque was defined according to the Mannheim consensus Single sonographer performed ultrasound
Jung [27]	Any	Examination by ophthalmologist	CCA IMT ≥ 1 mm, Plaques > 2	CCA IMT measured 1 cm proximal to bulb on left and right, mean of these were used Protrusions > 100% defined as plaque. Plaques were categorised into n ≤ 2 and n > 2 plaques
Cardoso [29]	Any	Examination by ophthalmologist	IMT of CCA, BIF, ICA Plaque score ≤ 2 or > 2	Mean of 3 IMT measurements used. IMT measured according to Mannheim consensus Extracranial carotid artery plaque score assigned for each segment of ICA, CCA and BIF and highest grade assigned; 0: no plaque, 1: one small plaque, stenosis < 30%, 2: one medium, plaque 30–49% stenosis or multiple small plaques, 3: one large plaque, 50–99% stenosis or multiple plaques with at least one medium plaque, 4: 100% occlusion Single vascular radiologist performed ultrasound, good intra-observer test–retest reliability
Yun [10]	Any	Retinal images graded according to ETDRS protocol	CCA IMT Plaque	CCA IMT measured by software at thickest point ~ 1 cm from bulb, analysed in tertiles Plaque defined as protrusions into lumen that were 100% thicker than surrounding area Physicians performed ultrasound
Son [10]	Any	Two-field retinal images and ophthalmologist examination	CCA IMT ≤ 0.9 mm and no plaque compared to CCA IMT > 0.9 mm ± carotid plaque	IMT measured bilaterally 5–10 mm proximal to bulb, 3 measurements done at site of greatest thickness and 10 mm proximal and distal to this point, highest mean CCA IMT used Plaque was a focal increase of ≥ 0.5 mm or ≥ 50% of surrounding IMT Single sonographer performed ultrasound
Araszkiewicz [30]	Any	Two-field retinal images and ophthalmologist examination according to American Academy of Ophthalmology	CCA IMT	Right CCA IMT measured and automatically calculated with software program—Carotid Analyzer for Research (CAD 5)
Lacroix [33]	Any	Examination by ophthalmologist	no atherosclerotic lesion or stenosis < 60% or stenosis ≥ 60%	Stenosis was considered ≥ 60% when the maximal velocity within the lesion was > 2.6 m/s and the end-diastolic velocity > 0.7 m/s Performed by experienced vascular physicians
Distiller [31]	Any	Retinal images assessed	CCA IMT Plaque IMT risk (low, medium or high)	CCA IMT measured > 1 cm proximal to flow divider, mean of left and right used Plaque defined as localised thickening of wall of ≥ 1.5 mm IMT risk: low < 0.6 mm, medium 0.6–0.8, high > 0.8 and/or plaque Two sonographers, intra-observer & inter-observer variability 3.1% and 3.9%
Rema [9]	Any	Four-field retinal images graded according to ETDRS protocol by two graders, a third grader made final decision if discrepancy	IMT of CCA, BIF, ICA	Mean of six IMT measurements of right ICA, CCA and BIF All scans were quality controlled by a central laboratory in Canada

*DR* diabetic retinopathy, *NPDR* non-proliferative diabetic retinopathy, *PDR* proliferative diabetic retinopathy, *CSMO* clinically significant macular oedema, *VTDR* vision-threatening diabetic retinopathy, *ETDRS* Early Treatment Diabetic Retinopathy Study, *CCA* common carotid artery, *ICA* internal carotid artery, *IMT* intima-media thickness

The methods, results and limitations of these studies are summarised in Table 3. Ten studies used carotid disease as the dependent variable in analysis [7, 10, 23, 27–29, 31–34] and the remaining four used DR as the dependent variable [8–10, 30]. However, as only one study had a longitudinal component (with carotid disease as dependent variable), the directionality of any association was not able to be determined. The results from the cross-sectional studies suggest that there is some association between the two complications (see Table 4). In general, there was a statistically significant relationship between one or more measure of carotid disease and DR. Four of the 14 studies reported no statistically significant association between any carotid disease variable and any DR variable [10, 23, 30, 32].

**Table 3:** Methods, limitations and results of relevant studies

First author	Methods	Results	Variables included in multivariate model	Limitations
Studies which used carotid disease as the dependent variable:				
Cardoso [23]	Tertiary-care university hospital outpatients were consecutively recruited Required to have either any microvascular complication or macrovascular complication with at least 2 modifiable risk factors Excluded if > 80 years old, BMI ≥ 40 kg/m^2^, serum creatinine ≥ 2 mg/dl or poor life expectancy Participants followed up till first endpoint or end of study Cox regression used	No measure of carotid disease was associated with new or worsening DR in most adjusted model From personal communication from author, for highest versus lowest tertile of IMT, the HR (95% CI) was 0.99 (0.59–1.64) *P *= 0.95 for CCA, 1.23 (0.73–2.06) *P *= 0.44 for BIF and 1.17 (0.71–1.93) *P *= 0.53 for ICA Carotid plaque score ≥ 3 points was also not significant; 1.69 (0.88–3.24) *P *= 0.12	Age, sex, diabetes duration, BMI, smoking, physical activity, clinic SBP, number of antihypertensive drugs, use of insulin and statins, presence of macrovascular diseases and baseline DR, mean HbA_1c_, HDL and LDL during first year of follow up	Selection bias—recruited from tertiary hospital clinic so likely complex type 2 diabetes participants Potential measurement bias as single ophthalmologist Unclear risk of attrition bias
Hjelmgren [32]	Participants were recruited from the Western Region initiative to Gather Information of Atherosclerosis (WINGA) database Participants who were referred for ultrasound after suffering first ischaemic stroke or TIA were consecutively included Excluded if < 40 years old, no ultrasound within 6 months of event or if information on DR ambiguous Logistic regression used	Any DR did not increase the odds of carotid stenosis (OR: 0.79 (0.48–1.30), *P *= 0.35)	Age, CHD, HF, PAD and creatinine	Selection bias—only included those who had experienced an ischaemic stroke or TIA Potential detection bias as information from medical records may not be complete and detection may vary between hospitals/clinics Used stenosis > 50% as outcome so would have missed lower rates of occlusion/CIMT increase Potential measurement bias—unsure about who/how many conducted the carotid ultrasound High proportion of males Limited generalisability
Carbonell [7]	Recruited from two outpatient university hospital clinics belonging to the same health care organisation Participants were identified from electronic clinical records and included if > 18 years old and diabetes duration ≥ 1 year Excluded if history of CVD, diabetic foot disease, eGFR < 60 ml/min/1.73 m^2^ or uACR > 300 mg/g Logistic and multinomial logistic regression used	Advanced DR (OR: 2.66 (1.03–6.95) *P* = 0.044) but not mild DR (1.35 (0.66–2.76) *P *= 0.41) was independently associated with carotid plaque Advanced DR (OR 4.71 (1.48–15.04) *P *= 0.009) was also independently associated with increased odds of ≥ 2 carotid plaques The presence of any DR was not statistically significantly associated with any plaque (1.64 (0.85–3.17) *P *= 0.14) or ≥ 2 plaques (1.93 (0.83–4.47) *P *= 0.129)	Age, sex, diabetes duration, smoking, diastolic BP, dyslipidaemia, uACR, BMI, pulse pressure and LDL	Selection bias—only recruited from clinics Excluded those with CVD and in doing so may have excluded some with carotid disease Sample size was calculated on the presence of DR and not advanced DR Potential measurement bias as single ophthalmologist and sonographer at each site
Liu [34]	Participants were from the Diabetes Health Management Program, a community-based system of electronic health records recruited via free health check-up annually for residents and household survey at Meilong Town Excluded if < 40 years of age or history of CVD Linear and logistic multiple regressions used	Any DR was associated with CCA IMT (mm) (coefficient 0.015, *P *= 0.010, Standard error: 0.080) in linear Regression Any DR was associated with CCA IMT > 1 mm (OR:1.84 (1.02–3.31) *P *= 0.043), presence of plaque (1.87 (1.03–3.39) *P *= 0.039) and subclinical atherosclerosis (1.93 (1.03–3.60) *P *= 0.039) in most adjusted logistic regression models	Age, sex, alcohol use and LDL in all models. The logistic regressions also adjusted for smoking, hypertension, diabetes duration, HbA_1c_, use of antidiabetic drugs, insulin use, antihypertensive drugs, obesity, Triglycerides, total cholesterol, HDL, eGFR, uACR and GGT	Excluded those with CVD and in doing so may have excluded some with carotid disease Potential measurement bias as single ophthalmologist and sonographer Unclear risk of selection bias
Alonso [28]	Recruited based on medical records from an outpatient clinic and diabetic eye disease program Tried to match those with DR and those without on age and sex Excluded those with CVD or impaired renal function General linear models used for IMT and logistic regression for plaque presence	Any DR was associated with mean ICA IMT (*P *= 0.0176) but not CCA IMT or bifurcation IMT Any DR increased odds of any plaque (OR: 1.71 (1.03–2.85) *P *= 0.0366) and the odds of ≥ 2 plaques (3.17 (1.75–5.75)) *P *< 0.0001)	All models adjusted for age. Also, in general linear models CCA IMT adjusted for smoking; bifurcation IMT for hypertension; and ICA IMT for sex. In logistic regression for any plaque adjusted for hypertension and smoking; and for ≥ 2 plaques for sex and dyslipidaemia	Selection bias—only recruited from clinics Excluded those with CVD and in doing so may have excluded some with carotid disease Potential measurement bias as single ophthalmologist and sonographer
Jung [27]	Hospital patients’ notes were retrospectively reviewed Excluded if malignancy, hepatic failure, acute infection, acute metabolic complications, fatal arrhythmia or CVD Logistic regression used	Any DR was independently associated with CCA IMT > 1 mm (OR: 3.8 (1.4–10.2)) but not > 2 carotid plaques (OR: 5.7 (0.6–51.3))	Age, diabetes duration, smoking, hypertension, HbA_1c_, cardiac autonomic neuropathy, brachial-ankle pulse wave velocity, statin use, ACE-I/ARB use and eGFR	Potential detection bias -retrospectively analysed medical notes which relies on all data being available/recorded appropriately All tests would have been done as part of usual diabetes care so may reflect a higher risk population Excluded those with CVD and in doing so may have excluded some with carotid disease Potential measurement bias—single ophthalmologist and limited detail on who/how many performed ultrasounds Relatively small, young sample Limited generalisability
Cardoso [29]	Tertiary -care university hospital outpatients were consecutively recruited Required to have either any microvascular complication or macrovascular complication with at least 2 modifiable risk factors Excluded if > 80 years old, BMI ≥ 40 kg/m^2^, serum creatinine ≥ 2 mg/dl or poor life expectancy Generalised linear models were used with DR as a fixed factor to assess relationship with IMT Logistic regression used to assess relationship between DR and plaque score	Any DR was associated with increased odds of a plaque score > 2 (OR: 1.70 (1.02–2.84) *P *= 0.043) Any DR was not independently associated with IMT at ICA, BIF or CCA in either logistic or linear regression but effect size and *P*-values not given	The logistic regression for plaque score adjusted for age, sex, smoking, antihypertensive use and aortic pulse wave velocity. All IMT linear and logistic regressions adjusted for age and night-time pulse pressure. Additionally, CCA IMT adjusted for sex, smoking and antihypertensive use; bifurcation IMT for LDL and smoking; and ICA IMT for sex and C-reactive protein in logistic regression as well as smoking in linear regression.	Potential selection bias—recruited from tertiary hospital clinic so likely complex type 2 diabetes participants Potential measurement bias as single ophthalmologist
Son [10]	Consecutive patients of an outpatient diabetes centre diagnosed with diabetes during the study period were recruited Excluded those with longer duration of diabetes, CVD or cerebrovascular events Logistic regression used	Any DR increased the odds of plaque or increased CCA IMT > 0.9 mm (OR: 6.57 (1.68–25.71) *P *= 0.007) compared to those with CCA IMT < 0.9 mm and no plaque	Age, sex, smoking, hypertension, BMI, diabetic nephropathy, HbA_1c_, fasting glucose, HDL and LDL	High proportion of males Only comprised participants with newly diagnosed diabetes Small sample size Excluded those with CVD and in doing so may have excluded some with carotid disease Potential measurement bias – single ophthalmologist and limited detail on who/how many performed ultrasounds
Lacroix [33]	Patients with diabetes referred to a vascular laboratory were consecutively recruited Excluded those with life expectancy > 12 months, a recent (< 6 weeks) stroke or TIA, carotid surgery, cervical radiotherapy or symptoms of carotid disease Multiple logistic regression used	Any DR increased odds of any carotid stenosis (2.38 (1.06–5.33) *P *= 0.03) Any DR increased odds of carotid stenosis ≥ 60% (3.62 (1.12–11.73), *P *< 0.0001) compared to no or < 60% stenosis	Age > 70 years, hypertension, BMI, history of CHD and family history of diabetes in model for any stenosis. Sex, ABI and history of ischemic neurological disorder or cervical bruit in model for stenosis ≥ 60%	Selection bias—recruited from referrals to a specialist clinic and excluded those with symptoms of carotid disease Study was focussed on screening for carotid disease Potential measurement bias—limited detail on who/how many people performed ophthalmic exams or ultrasounds
Distiller [31]	Patients with diabetes were recruited from the Centre for Diabetes and Endocrinology Included those with at least 10 measurements of Hba_1c_ in last 5 years, normal renal function, no proteinuria Those on statins > 5 years, with an underlying autoimmune disease, nephropathy, on steroids or those with hypothyroidism with inadequate replacement were excluded Multiple logistic regression, linear regression and ordinal logistic regression used	Any DR increased the odds of plaque; OR: 3.65 (1.11–12.02) *P *= 0.033, but not IMT or IMT risk (effect for these not given)	In multiple regression for IMT adjusted for age, diabetes duration, BMI, hypertension and HDL. In ordinal regression for IMT risk adjusted for age, triglyceride:HDL ratio and HbA_1c_. In logistic regression for plaque adjusted for age, hypertension and smoking	Some selection bias as recruited from a diabetes centre and only Caucasians with long diabetes duration Potential measurement bias—limited information given on ascertainment of DR status

First author	Methods	Results	Results adjusted for	Limitations
Studies which used diabetic retinopathy as the dependent variable:				
Ichinohasama [8]	Unclear how participants recruited, but underwent assessment at hospital Excluded those with HbA_1c_ < 6.5 and if not on ongoing diabetes therapy, those on haemodialysis, or who had malignancy, inflammatory disease, chronic respiratory disease, macular degeneration, or glaucoma or other retinal disease Logistic regression analysis used	CCA IMT increased the odds of mild NPDR (OR: 8.65 (1.95–38.4) *P *= 0.005, per 1 mm increase)	Age, sex, duration of diabetes, HbA_1c_ diastolic blood pressure, heart rate, creatinine, central macular thickness and mean blur rate in the overall optic nerve head	Potential selection bias—participant recruitment was not clear Only right sided IMT and DR assessed Participants had no or mild DR, none with more severe DR Excluded those with T2D with HbA_1c_ < 6.5 and diet controlled Potential measurement bias as single ophthalmologist and sonographer
Yun [10]	Participants registered at a public health centre who had participated in another survey were recruited Excluded those with missing data including blood, urine, HbA_1c_, diabetes duration, CCA-IMT, carotid plaque, baPWV, DR outcome Logistic regression analysis used	CCA IMT was not associated with DR in most adjusted model (OR for tertile 2: 1.16 (0.67–2.02) and tertile 3: 1.06 (0.59–1.90) when compared to tertile 1, P = 0.844) Carotid plaque was not associated with DR (OR: 1.20 (0.75–1.91))	Age, sex, duration of diabetes, HbA_1c_, total cholesterol, triglycerides, HDL, eGFR, BMI and history of hypertension	High proportion of females Potential measurement bias—unclear about who graded DR and the reliability and validity of the ultrasounds
Araszkiewicz [30]	Hospital patients admitted for diabetes management recruited consecutively Excluded those > 50 years old, liver dysfunction, chronic kidney disease ≥ stage 3, anaemia, acute inflammation, CVD, CHD, PVD, DKA on admission or carotid stenosis > 50% Logistic regression analysis performed	CCA IMT not associated with DR in multivariate analysis (OR: 1.00 (0.99–1.01) P = 0.169 per 1 μm increase)	Age, sex, diabetes duration, albuminuria, BP, postprandial glucose, HbA_1c_, central augmentation index and peripheral augmentation index	Potential selection bias—recruited from a hospital Only right sided IMT measured Likely excluded those with higher IMT as excluded if stenosis > 50% and CVD Small numbers Potential measurement bias as single ophthalmologist and unclear who performed ultrasounds
Rema [9]	A random sample of 450 participants with known and 150 participants with newly diagnosed diabetes from a population-based study were assessed Multivariate regression models were used	Mean IMT increased the odds of DR (OR: 2.9 (1.17–7.33), P = 0.024 per 1 mm increase)	Age, HbA_1c_, duration of diabetes and microalbuminuria	Unclear selection bias risk Only used the right carotid ultrasound

*DR* diabetic retinopathy, *VTDR* vision threatening diabetic retinopathy, *IMT* intima-media thickness, *CCA* common carotid artery, *ICA* internal carotid artery, *BIF* bifurcation, *CVD* cardiovascular disease, *uACR* urinary albumin:creatinine ratio, *CHD* coronary heart disease, *PVD* peripheral vascular disease, *DKA* diabetic ketoacidosis, *TIA* transient ischaemic attack, *HF* heart failure, *PAD* peripheral arterial disease, *BP* blood pressure, *BMI* body mass index, *LDL* low density lipoprotein, *HDL* high density lipoprotein, *eGFR* estimated glomerular filtration rate, *GGT* gamma-glutamyl transferase, *ACE-I* angiotensin converting enzyme inhibitors, *ARB* angiotensin receptor blockers, *ABIs* ankle-brachial index

**Table 4:** Summary showing which studies reported significant and non-significant associations between carotid disease and retinopathy

Carotid disease variable		Any diabetic retinopathy	Other diabetic retinopathy
CCA IMT	Non-significant	Alonso [28] Cardoso [29] Distiller [31] Yun [10] Araszkiewicz [30]	Alonso [28]—DR severity Cardoso [23]— new or worsening DR
	Significant	Liu [34] Jung [27]	Ichinohasama [8]—mild NPDR
Other IMT	Non-significant	Alonso [28]—BIF Cardoso [29]—ICA & BIF	Alonso [28]—ICA & BIF, DR severity Cardoso [23] —ICA & BIF, new or worsening DR
	Significant	Alonso [28]—ICA Rema [9]—Mean IMT	
Plaque/Stenosis	Non-significant	Hjelmgren [32] Carbonell [7] Jung [27] Yun [10]	Alonso [28]—DR severity Cardoso [23]—new or worsening DR
	Significant	Liu [34] Alonso [28] Lacroix [33] Distiller [31] Cardoso [29]	Carbonell [7]—advanced DR
Other	Non-significant	Distiller [31]—IMT risk	
	Significant	Liu [34]—any plaque and/or CCA IMT > 1 mm Son [10]—any plaque and/or CCA IMT > 0.9 mm	

*BIF* bifurcation, *CCA* common carotid artery, *DR* diabetic retinopathy, *ICA* internal carotid artery, *IMT* intima-media thickness

The four main carotid variables identified were CCA IMT, other measures of IMT including combinations with CCA IMT, plaque or stenosis, and other carotid disease that mostly comprised a combination of carotid variables. Half of the studies assessed only one carotid disease variable [7–10, 30, 32, 33] while the other half assessed more than one carotid disease variable [10, 23, 27–29, 31, 34]. Of the seven studies which assessed multiple such variables, three found a significant relationship between DR and carotid plaque only [28, 29, 31], one reported an association with CCA IMT only [27], two reported no significant association [10, 23] and one reported a significant association with all carotid variables assessed [34]. Of all included studies, two reported that DR was significantly associated with a combined measure of plaque and IMT [10, 34]. Nine of the 14 papers specifically assessed the relationship with plaque/stenosis and DR and six reported a significant association [7, 28, 31, 33, 34]. Plaque was often significantly associated with carotid disease, but this finding was not consistent across all studies.

Ten studies assessed the relationship with CCA IMT and DR. Three of these found a significant association, two reported CCA IMT was associated with any DR [27, 34] and one reported an association with mild NPDR [8]. Interestingly, the two studies that reported an association between any DR and CCA IMT used a cut-off point for CCA IMT of ≥ 1 mm [27, 34] while those that reported no association assessed CCA IMT as a continuous variable or split the IMT by tertiles [10, 28–31]. Some of these studies may have had insufficient statistical power, especially as one comprised just 87 participants [30], and only one provided a sample size calculation [28]. Four studies assessed other measures of IMT, of which one determined an association between DR and internal carotid artery (ICA) IMT [28] and one reported that the mean IMT of the ICA, CCA and bifurcation was associated with DR [9]. There did not appear to be a single marker of carotid disease that consistently identified an increased likelihood of DR presence or severity. However, most studies have shown that there is a relationship between at least one measure of carotid disease and DR.

While most studies showed a significant association between carotid disease and DR, four studies reported no significant relationship. Three of these were of good quality [10, 23, 30], while one was of poor quality [32]. The study by Araszkiewicz et al. had a small sample size compared to the other studies and excluded those with carotid stenosis > 50%; this probably also excluded those with a high common carotid artery (CCA) IMT which may explain the lack of association [30]. Hjelmgren et al. assessed the relationship with stenosis and defined stenosis as a narrowing of > 50%, excluding less severe cases [32]. In the study by Yun et al., CCA IMT was analysed by tertiles, the highest of which ranged from 0.79 to 1.30 mm, including those without clinically significant intima-media thickening of ≥ 1 mm [10]. However, this study also assessed the relationship with carotid stenosis and found no significant association [10]. The longitudinal RDJ study found no statistically significant association between any measure of carotid disease and new or worsening DR, but this sample was outpatient clinic-based [23]. The lack of association seen in some studies may be due to methodological differences.

A common limitation was that most studies used convenience sampling and recruited participants from hospitals and clinics. This increases the likelihood of selection bias, as these participants are more likely to be complex and therefore not representative of the general population of people with diabetes. There was, however, one population-based study [9] and one study that recruited participants from a community-based health program [34]. Measurement bias for DR and carotid disease was another limitation in many studies, with few studies reporting on inter- or intra-rater agreement or quality control measures. Other limitations and potential sources of bias included assessment of only one eye or one carotid artery, and exclusion of participants with a history of cardiovascular disease and thus at relatively high risk of carotid disease. Many studies did not report their response rate. The quality of the individual studies is shown in Additional file 1: Table S1. Overall, five studies were of poor quality [8, 30–33], one fair quality [27] and the remaining eight were of good quality [7, 9, 10, 23, 28, 29, 34]. There were no conflicts of interest identified in any of the studies.

## Discussion

This is the first systematic review to assess the relationship between carotid arterial disease and DR. In most included studies there was a statistically significant association between at least one carotid disease parameter and DR presence or severity as assessed from either an increased risk of DR in the presence of carotid disease or an increased risk of carotid disease in people with known DR. No specific carotid disease variable was consistently associated with DR. Although this may reflect significant methodological differences between the studies, most that assessed the relationship between carotid stenosis/plaque and DR reported a significant association. In addition, as only one study utilised longitudinal data and found no association, the directionality of this relationship remains unknown.

There is evidence that macrovascular disease is associated with the microvascular complications of diabetes. A recent meta-analysis has shown that DR is associated with an increased risk of stroke [35]. Diabetic retinopathy has also been shown to increase cardiovascular disease risk [36], and the severity and progression of DR was associated with cardiovascular disease in the ACCORD study [37]. A significant association between carotid disease and changes in the retinal microvasculature has also been found in adolescents with T1D [38]. Diabetic retinopathy has also been associated with other markers of peripheral arterial disease [39], peripheral vascular disease in T1D [40], and arterial stiffness [41].

The mechanisms underlying the relationship between carotid disease and DR are not well established, although there are several theories. First, the “common soil” hypothesis suggests that microvascular and macrovascular complications share risk factors including hyperglycemia, dyslipidemia and hypertension [19, 29, 42]. The Hoorn Diabetes Care System cohort study also showed that glycaemic variability was associated with both microvascular and macrovascular disease [43]. Second, it has been suggested that signs of microvascular damage in the eye could reflect concurrent microcirculatory disease in the heart and major arteries [36]. One study found that the presence of DR was associated increased neovascularisation in the vasa vasorum of the carotid artery, although there were no differences in IMT or carotid plaque by DR status [44]. Nevertheless, increased neovascularisation of the vasa vasorum has been associated with atherosclerosis and plaque in another study [45]. Although microvascular damage may be a potent underlying cause of macrovascular disease [46], we cannot evaluate this from the studies included in this review as the only longitudinal study assessed whether carotid disease was associated with new or worsening DR, and thus whether macrovascular disease affected microvascular complications.

Lastly, proximal macrovascular disease may directly affect the microvasculature. Interestingly, almost half of the studies in this review excluded participants with known cardiovascular disease, which is a common complication of diabetes. Of these, most reported a significant association between DR and carotid disease [7, 10, 27, 28, 34], although one found no association [30]. As there were still statistically significant associations in the absence of known cardiovascular disease, that there may be more to the relationship between carotid disease and DR than simply long term exposure to shared risk factors, supporting a direct relationship between carotid disease and DR.

The presence of stenosis or plaque was more often significantly associated with DR than other measures of carotid disease. As the carotid artery supplies the ophthalmic artery, it is possible that microemboli or plaque fragments from the carotid could dislodge and travel to the retina [47] thus contributing to the microvascular occlusion and ischaemia that occur in DR. Alternatively, carotid stenosis has a greater effect on the blood flow through the carotid artery than thickening measured by IMT. It is plausible that significant stenosis causing disruption of carotid blood flow could impact the blood supply to the retina. Improved retinal blood flow assessed by optical coherence tomography angiography has been reported in participants with carotid stenosis > 70% after carotid endarterectomy [48], and there is evidence that vision improves after carotid endarterectomy [49]. However, as some studies found that IMT was independently associated with DR, more than one mechanism is likely with a combination of common soil and direct relationships.

## Conclusions

We found that there is insufficient published evidence to determine the nature, including the directionality, of the relationship between DR and carotid disease. Well conducted longitudinal studies are required to determine the direction of this association. A significant association was reported in most evaluable studies, but whether these two complications are independent but co-exist or if one contributes to the other can only be determined by well-conducted longitudinal studies. Nevertheless, our findings have clinical implications. Should carotid atherosclerosis or stenosis be detected in an individual with diabetes, we recommend that retinal examination is performed and, conversely, intensified cardiovascular risk management should be considered if DR is detected.

## Supplementary information

**Additional file 1: Figure S1.** Diagram of a carotid artery with plaque at the bifurcation. **Table S1.** The quality of the included studies.

## Data Availability

Not applicable.

## Abbreviations

ACCORD: Action to Control Cardiovascular Risk in Diabetes
CCA: Common carotid artery
DR: Diabetic retinopathy
FIELD: Fenofibrate Intervention and Event Lowering in Diabetes
ICA: Internal carotid artery
IMT: Intima media thickness
NPDR: Non-proliferative diabetic retinopathy
PRISMA: Preferred Reporting Items for Systematic Review and Meta-Analysis
RDJ: Rio de Janiero Type 2 Diabetes Cohort Study
T1D: Type 1 diabetes
T2D: Type 2 diabetesVEGF- vascular endothelial growth factor
