# Supplementary material for: The relationship between carotid disease and retinopathy in diabetes: a systematic review
Source: Cardiovasc Diabetol. 2020 May 6;19:54. doi: 10.1186/s12933-020-01023-6 (PMC7201797; doi:10.1186/s12933-020-01023-6)
Supplement: Supplementary file 1 — Additional file 1: Figure S1. Diagram of a carotid artery with plaque at the bifurcation. Table S1. The quality of the included studies. [file 12933_2020_1023_MOESM1_ESM.docx]

**Additional Material**

Figure S1: Diagram of a carotid artery with plaque at the bifurcation

Degree of stenosis – the percentage of artery narrowing

Internal carotid artery

Bifurcation

Common carotid artery

Intima media thickness

External carotid artery

Plaque

Table S1: The quality of the included studies

|  | | Studies with carotid disease as dependent variable | | | | | | | | | | Studies with retinopathy as dependent variable | | | |
| --- | --- | --- | --- | --- | --- | --- | --- | --- | --- | --- | --- | --- | --- | --- | --- |
| First author: | | Cardoso, 2019 ^21^ | Hjelmgren ^31^ | Carbonell ^6^ | Liu ^33^ | Alonso ^27^ | Jung ^25^ | Cardoso ^28^ | Son ^26^ | Lacroix ^32^ | Distiller ^30^ | Ichinohasama ^7^ | Yun ^9^ | Araszkiewicz ^29^ | Rema ^8^ |
| Question number of NHLBI quality tool for observational  cohort and cross-sectional studies | 1 | Yes | Yes | Yes | Yes | Yes | Yes | Yes | Yes | Yes | Yes | Yes | Yes | Yes | Yes |
|  | 2 | Yes | Yes | No | Yes | No | Yes | Yes | Yes | No | No | No | No | No | No |
|  | 3 | NR | Yes | NR | Yes | NR | NR | NR | NR | Yes | NR | NR | Yes | Yes | NR |
|  | 4 | Yes | Yes | Yes | Yes | Yes | Yes | Yes | Yes | Yes | Yes | NR | Yes | Yes | Yes |
|  | 5 | No | No | Yes | No | Yes | No | No | No | No | No | No | No | Yes | No |
|  | 6 | Yes | No | No | No | No | No | No | No | No | No | No | No | No | No |
|  | 7 | Yes | No | No | No | No | No | No | No | No | No | No | No | No | No |
|  | 8 | No | No | Yes | No | Yes | No | No | No | No | No | Yes | Yes | No | Yes |
|  | 9 | Yes | CD | Yes | Yes | Yes | Yes | Yes | Yes | Yes | CD | Yes | Yes | Yes | Yes |
|  | 10 | Yes | No | No | No | No | No | No | No | No | No | No | No | No | No |
|  | 11 | Yes | CD | Yes | Yes | Yes | Yes | Yes | Yes | CD | Yes | Yes | Yes | Yes | Yes |
|  | 12 | Yes | CD | CD | CD | Yes | CD | Yes | Yes | CD | CD | CD | CD | CD | CD |
|  | 13 | CD | N/A | N/A | N/A | N/A | N/A | N/A | N/A | N/A | N/A | N/A | N/A | N/A | N/A |
|  | 14 | Yes | Yes | Yes | Yes | Yes | Yes | Yes | Yes | Yes | Yes | Yes | Yes | Yes | No |
| Overall: | | Good | Poor | Good | Good | Good | Fair | Good | Good | Poor | Poor | Poor | Good | Good | Poor |

Abbreviations: CD: cannot determine, NR: not reported, N/A: not applicable
